# Metabolomics datasets in the Born in Bradford cohort

**DOI:** 10.12688/wellcomeopenres.16341.1

**Published:** 2020-11-05

**Authors:** Kurt Taylor, Nancy McBride, Neil J Goulding, Kimberley Burrows, Dan Mason, Lucy Pembrey, Tiffany Yang, Rafaq Azad, John Wright, Deborah A Lawlor

**Affiliations:** 1 Population Health Science, Bristol Medical School, University of Bristol, Bristol, BS8 2BN, UK; 2 MRC Integrative Epidemiology Unit, University of Bristol, Bristol, BS8 2BN, UK; 3 Bristol NIHR Biomedical Research Centre, University of Bristol, Bristol, BS1 2NT, UK; 4 Bradford Institute for Health Research, Bradford Hospitals National Health Service Trust, Bradford, BD9 6RJ, UK; 5 Department of Medical Statistics, London School of Hygiene & Tropical Medicine, London, UK; 6 Department of Biochemistry, Bradford Royal Infirmary, Bradford, UK; 7 Wolfson Centre for Applied Health Research, Bradford Hospitals National Health Service Trust, Bradford, BD9 6RJ, UK

**Keywords:** Metabolomics, mass spectrometry, nuclear magnetic resonance, pregnancy, mother, offspring health

## Abstract

Metabolomics is the quantification of small molecules, commonly known as metabolites. Collectively, these metabolites and their interactions within a biological system are known as the metabolome. The metabolome is a unique area of study, capturing influences from both genotype and environment. The availability of high-throughput technologies for quantifying large numbers of metabolites, as well as lipids and lipoprotein particles, has enabled detailed investigation of human metabolism in large-scale epidemiological studies. The Born in Bradford (BiB) cohort includes 12,453 women who experienced 13,776 pregnancies recruited between 2007-2011, their partners and their offspring. In this data note, we describe the metabolomic data available in BiB, profiled during pregnancy, in cord blood and during early life in the offspring. These include two platforms of metabolomic profiling: nuclear magnetic resonance and mass spectrometry. The maternal measures, taken at 26-28 weeks’ gestation, can provide insight into the metabolome during pregnancy and how it relates to maternal and offspring health. The offspring cord blood measurements provide information on the fetal metabolome. These measures, alongside maternal pregnancy measures, can be used to explore how they may influence outcomes. The infant measures (taken around ages 12 and 24 months) provide a snapshot of the early life metabolome during a key phase of nutrition, environmental exposures, growth, and development. These metabolomic data can be examined alongside the BiB cohorts’ extensive phenotype data from questionnaires, medical, educational and social record linkage, and other ‘omics data.

## Introduction

Metabolomics is the quantification of small molecules resulting from metabolic processes. The metabolome is influenced by both genotype and environment, and dynamically responds to environmental influences. Developments in high-throughput technologies have allowed the efficient and accurate quantification of metabolites. This has revolutionised our ability to understand the causes and consequences of variation in human metabolism, and the contribution that multiple metabolites can make to risk prediction, using large-scale epidemiological studies ^1– 4^. Lipids and lipoproteins, which are measured in most high-throughput platforms used in epidemiology, are larger than the threshold used to define metabolites (<1.5k Daltons) and are therefore metabolomic traits. For simplicity in this paper we refer to these as metabolites.

Birth cohorts can be useful for exploring prenatal influences on birth and later life outcomes. Recently, studies have shown metabolomic profiling can aid us in our understanding of maternal health during pregnancy ^3– 5^ and of the influence of *in utero* exposures on subsequent offspring health ^6, 7^. The Born in Bradford (BiB) study is a UK longitudinal birth cohort ^8^. Nuclear magnetic resonance (NMR) and mass spectrometry (MS) data are available in BiB including measurements during pregnancy, cord blood and early life in the offspring. MS offers a truly untargeted approach with comprehensive coverage of the metabolome (>1,000 metabolites) due to its high sensitivity. However, MS only provides relative quantification based on peak area in these approaches without comparison to a metabolite reference standard. NMR offers less coverage of the metabolome, but with absolute quantification possible in clinically meaningful units (e.g. mmol/L).

**Figure 1.**
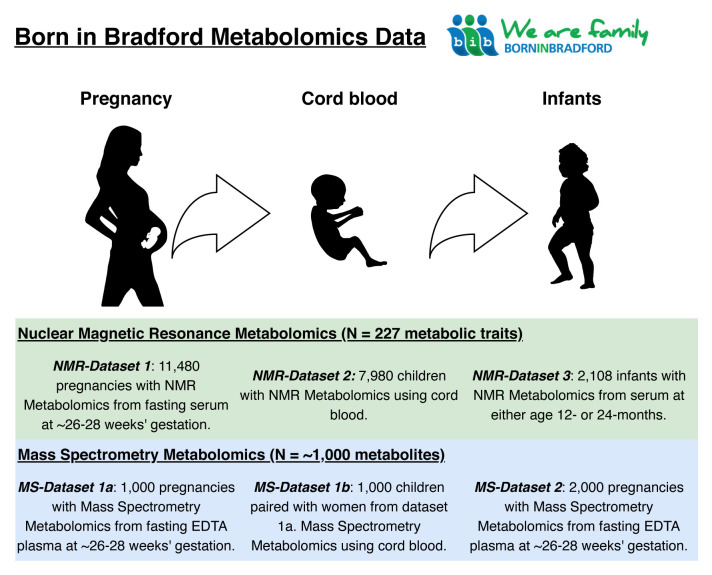
Summary illustration of the Born in Bradford metabolomics data. NMR, nuclear magnetic resonance; MS, mass spectrometry; EDTA, ethylenediaminetetraacetic acid.

The range of metabolomics data in BiB, coupled with the substantial data obtained through questionnaires, research clinic assessments, linkage to medical records, educational and social records, genome wide (mothers, offspring and a subgroup of fathers) and epigenome wide (mother and offspring) profiling makes BiB a valuable resource for metabolomics research. This data note describes the metabolomics data currently available in BiB - how these were obtained, quantified, utilised, as well as potential future uses, strengths and limitations. Figure 1 provides an illustrative summary of which type of metabolomic data have been collected on which cohort participants and when, up to 2020. Planned further metabolomic data collection is also described (see *Using the BiB metabolomic data*).

## Methods

### Ethical approval and consent

Ethical approval for the study was granted by the Bradford National Health Service Research Ethics Committee (ref 06/Q1202/48), and all participants gave written informed consent. The ALL IN sub-study had ethical approval from the London School of Hygiene & Tropical Medicine ethics committee (ref: 5320) and the Bradford Research Ethics committee (ref: 08/H1302/21). Parents (usually the mother) gave informed, written consent to take part in the study.

### Cohort

The BiB study is a population-based prospective birth cohort. In total, 12,453 women who experienced 13,776 pregnancies were recruited at their oral glucose tolerance test (OGTT) at approximately 26–28 weeks’ gestation, which was offered to all women booked for delivery at Bradford Royal Infirmary (BRI). Eligible women had an expected delivery between March 2007 and December 2010. The study is unique because it includes high proportions of White European and South Asian families, all residing in Bradford, UK. Bradford is a city in the North of England with high levels of socioeconomic deprivation, and the cohort was started due to a high prevalence of poor child health in the city ^8^. Full details of the study methodology were reported previously ^8^. The study website provides more information, including protocols, questionnaires and information on how researchers can access data and a full list of all available data. Mothers and their partners, who were recruited into the study, provided detailed interview questionnaire data, measurements, and biological samples. They also consented to the linkage of their and their child’s data to routine (primary and secondary care) health and education data.

### Blood sampling

Maternal overnight-fasted blood was taken during the OGTT and processed and stored at -80°C for further research and analyses. Infant cord blood samples were taken whenever possible (i.e. so long as staff were available, and collection of an umbilical vein sample did not interfere with care of the mother or infant) and immediately processed and stored at -80°C. Samples were taken in a subgroup of offspring in early childhood for a specific project on childhood viral infection ^9^. We describe the processes of taking, processing, and storing samples at each time point before moving on to describe the NMR and MS metabolomic profiling.

***Pregnancy blood samples.*** Of the 13,776 pregnancies in the BiB cohort, 11,480 had a fasting blood sample taken during the OGTT (n = 10,574 [92%] between 26–28 weeks’ gestation, with the remaining women being within 11–39 weeks’ gestation). Samples were taken by trained phlebotomists working in the antenatal clinic of the BRI and sent immediately to the hospital laboratory.

Venous blood was collected in GEL tubes to obtain serum and plasma. The following processing steps were undertaken prior to storage at -80°C.

1) Storage racks were prepared.
2) Participant details were checked, making sure that both the BiB study ID and hospital number on the specimen bottles matched those on the participant tracking forms.
3) Tubes were centrifuged at 3500 rpm for 10 minutes at room temperature.
4) A 1 ml automatic pipette was used to aliquot samples into 2 × 1.5 ml vials.
5) Vials were labelled with appropriate BiB study labels and the duplicate barcode label was placed in the corresponding space marked on BiB tracking form.
6) Aliquots (n = 3) were then placed in racks in a -80°C freezer.

All samples were processed within 2.5 hours and then placed in −80°C freezers. There were no freeze-thaw events of the samples prior to their use for the pregnancy metabolomic profiling. Serum samples were used for NMR metabolomic profiling, except for five (0.04%) samples which were plasma. For MS pregnancy metabolomics, ethylenediaminetetraacetic acid (EDTA) (a sample tube anticoagulant) plasma samples were used. Previous work has shown that reproducibility in both serum and plasma is good. As long as the same blood sample procedures are used (as in BiB), either matrix should yield similar results ^10^.

***Cord blood samples.*** Venous cord blood samples were all obtained at delivery by the attending midwife at the BRI, following research protocols. Cord blood sampling was not attempted for women delivering outside of the BRI, if the attending midwife was too busy, or if attempting to collect a research cord blood sample would interfere with postnatal care. Samples were refrigerated at 4°C in EDTA tubes until collected by BRI laboratory staff within 12 hours. Samples were then spun, frozen and stored at −80°C. In total, the BiB study collected 9,604 cord blood EDTA plasma samples. There were no freeze-thaw events of the cord blood samples.

***Infant blood samples.*** Infant metabolomics were performed on blood samples that were collected on a subsample of the BiB cohort; those enrolled into the Allergy and Infection Study (ALL IN) ^9^. Children enrolled in the BiB cohort, and born on or after 1 March 2008 with a maternal baseline questionnaire were eligible to take part in ALL IN. Mothers were invited to participate in ALL IN one month before their child’s first birthday. A questionnaire was completed by those who consented, and a 5ml venous blood sample was taken from the child, centrifuged, and stored at -80°C. This was repeated one year later to provide questionnaire data and serum from a ~12-month visit (mean age of 14 months, ranging from 9–18 months) and a ~24-month visit (mean age of 26 months, ranging from 23–33 months). Trained community research administrators (CRAs) recruited participants, obtained consent, and collected data, including blood samples, at each visit. They received training in phlebotomy and were assessed by the senior paediatric phlebotomist at the BRI. Ametop cream or Cryogesic spray were used to anaesthetise the venepuncture site. Only two attempts at venepuncture were permitted for each child. There was a fridge in the clinic for storing bloods before transfer to the lab. The blood samples taken on home visits were kept in a cool bag with an ice pack and then taken straight to the laboratory at BRI within 1–2 hours. The times of each step (blood taken, arrived at lab, centrifuged, aliquoted, frozen) were recorded on the blood form and were entered onto a database (so that researchers can check distribution of times if needed). For home or clinic visits outside normal working hours, the CRA who took the blood sample would centrifuge the blood at the lab and leave it in the lab fridge for processing the next day. All infant metabolomics were performed on serum samples. There was a maximum of two freeze-thaw events prior to metabolomics analyses of the infant samples.

### Metabolomic datasets in BiB

There are six metabolomics datasets including different populations and timepoints available in BiB. These are described below and summarized in Table 1 and Figure 1. We have divided the methods between the two main platforms (NMR and MS). We describe the methods used to generate each dataset and use flow charts to illustrate how selection was performed.

**Table 1.** Metabolomics datasets in the BiB cohort separated by platform.

#	Data source	Brief description
*Nuclear magnetic resonance*		
1	Pregnancy NMR – Dataset 1	N = 11,480 pregnancies. Single timepoint using maternal serum taken from a fasted blood sample around 26–28 weeks’ gestation. Of the 11,480, 37% are White British (40% White European) mothers and 44% Pakistani (49% South Asian).
2	Cord blood NMR – Dataset 2	N = 7,980 children. Single timepoint using cord blood, EDTA plasma.
3	Infants NMR (aged 12 or 24 months) – Dataset 3	N = 2,108 at either 12- or 24-months using serum samples. N = 1,690 at 12 months. N = 1,536 at 24 months. N = 1,118 at both timepoints.
*Mass Spectrometry*		
4	Pregnancy MS – Dataset 1a	N = 1,000 pregnancies. Single timepoint using EDTA plasma taken from a fasted blood sample between 26–28 weeks’ gestation. Of the 1,000, 50% are White British and the other 50% are Pakistani ethnicity.
5	Cord blood MS – Dataset 1b	N = 1,000 children (paired with women from Dataset 1a). Single timepoint using cord blood, EDTA plasma.
6	Pregnancy MS – Dataset 2	N = 2,000 pregnancies within a case-cohort design. EDTA plasma sample taken between 26–28 weeks’ gestation. Of the 2,000 women, 47% are White British and 53% are Pakistani.

NMR, nuclear magnetic resonance; MS, mass spectrometry; EDTA, ethylenediamine tetraacetic acid.

### NMR metabolomics

***NMR methods.*** We describe the NMR methods which apply to all the NMR datasets described in Table 1. Profiling of circulating lipids, fatty acids, and metabolites was done by a high-throughput targeted NMR platform (Nightingale Health© (Helsinki, Finland)) at the University of Bristol, providing quantitative information on 227 metabolic traits (including ratios and other traits derived from the quantified NMR spectra) ^1^. Details of all 227 traits can be found in the *Extended data* ^11^.

The Nightingale NMR metabolite quantification was achieved through measurements of three molecular windows from each serum/plasma sample. Two of the spectra (LIPO and LMWM windows) are acquired from native serum/plasma and one spectrum from serum lipid/plasma extracts (LIPID window). The NMR spectra are measured using Bruker AVANCE III spectrometer operating at 600 MHz. Measurements of native serum/plasma samples and serum/plasma lipid extracts are conducted at 37°C and 22°C, respectively.

The NMR spectra were analysed for metabolite quantification (molar concentrations) in an automated fashion. For each metabolite, a ridge regression model was applied for quantification to overcome the problems of heavily overlapping spectral data. In the case of the lipid data, quantification models were calibrated using high-performance liquid chromatography methods, and individually cross-validated against NMR-independent lipid data. Low-molecular-weight metabolites, as well as lipid extract measures, were quantified as mmol/L based on regression modelling calibrated against a set of manually fitted metabolite measures. The calibration data were quantified based on iterative line-shape fitting analysis using PERCH NMR software (PERCH Solutions Ltd., Kuopio, Finland). Quantification could not be directly established for the lipid extract measures due to experimental variation in the lipid extraction protocol. Therefore, serum/plasma lipid extract were scaled to total a standard serum cholesterol sample from the LIPO spectrum.

***Validation of the NMR platform.*** Quality control (QC) of the data were undertaken by Nightingale Health© prior to returning metabolite concentrations to BiB. Their QC procedures check various issues related to the sample integrity and the biomarker quantification. QC reports for the NMR datasets can be found in the *Extended data* ^11^.

**Figure 2.**
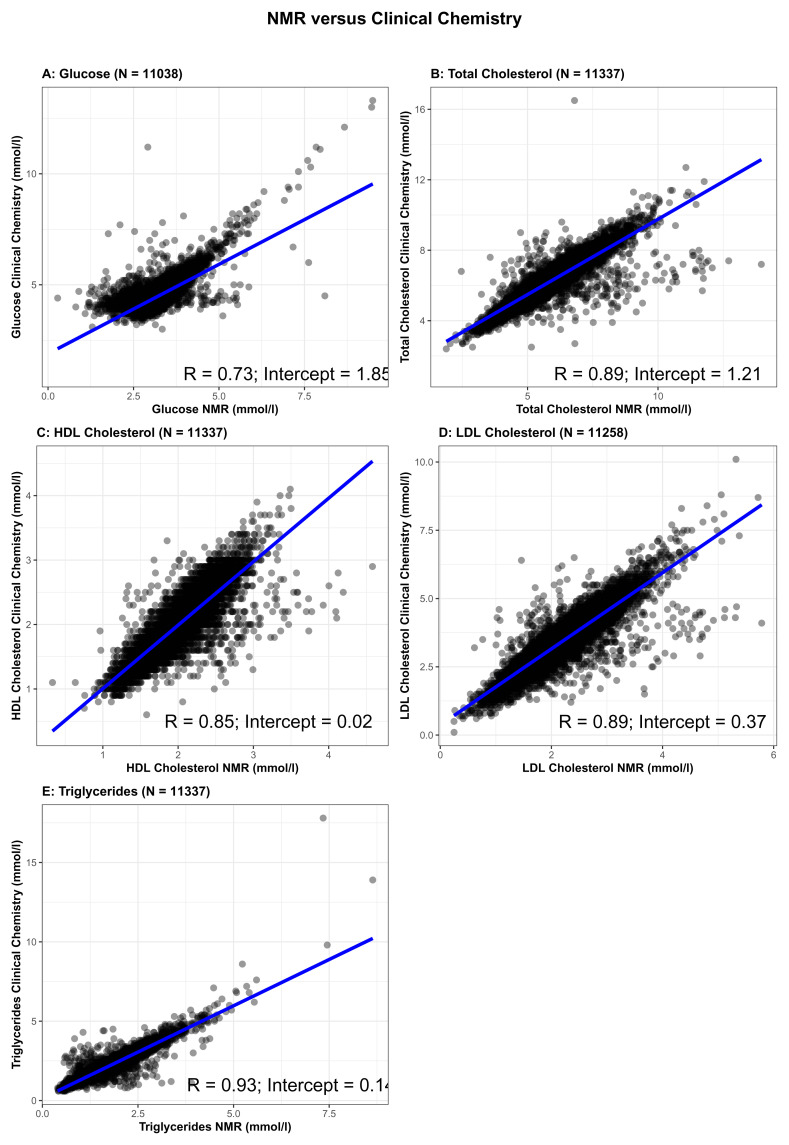
Comparison of glucose ( **2A**), total cholesterol ( **2B**), high-density lipoprotein cholesterol ( **2C**), low-density lipoprotein cholesterol ( **2D**) and triglycerides ( **2E**) concentrations between Nightingale Health© nuclear magnetic resonance (NMR) (x-axis) and routine clinical chemistry assays (y-axis) (N= 11,036 to 11,337). R = Pearson correlation coefficient.

**Figure 3.**
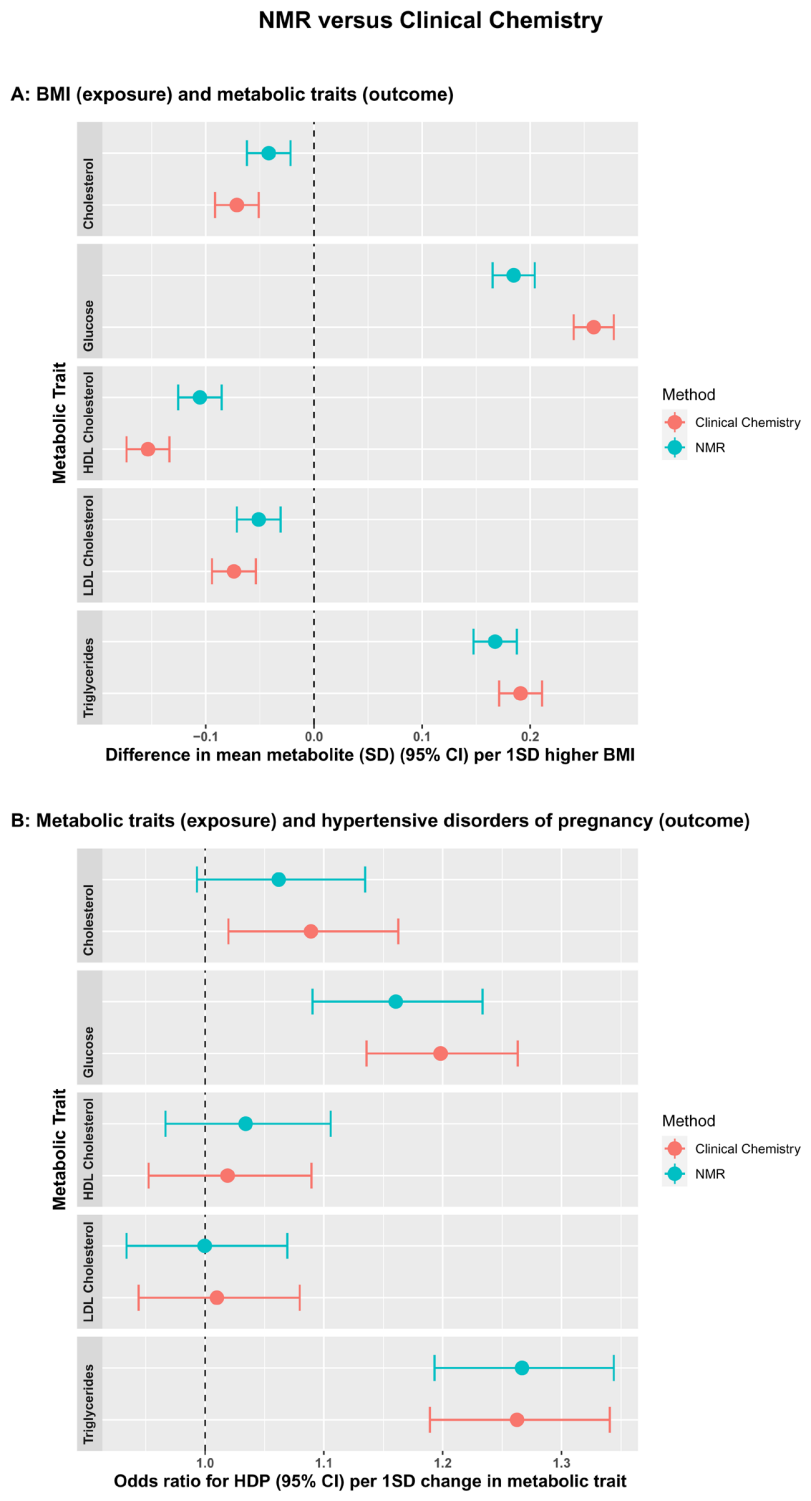
Comparisons of the associations of early pregnancy body mass index (BMI) with fasting glucose and lipids measured by Nightingale Health© nuclear magnetic resonance (NMR) and routine clinical chemistry assays ( **3A**), fasting glucose and lipids measured by Nightingale Health© NMR and routine clinical chemistry assays with hypertensive disorder of pregnancy (HDP) ( **3B**). Associations in **3A** are from unadjusted linear regression and data points show standard deviation differences in mean metabolite per one standard deviation (1SD) higher BMI. Associations in **3B** are from unadjusted logistic regression and data points show unadjusted odds ratios for HDP per 1SD higher in metabolic trait. Error bars = 95% confidence intervals.

We also undertook validation of some of the NMR measures by comparing concentrations of fasting glucose, total cholesterol, high-density lipoprotein cholesterol (HDLc), low-density lipoprotein cholesterol (LDLc), and triglycerides from the NMR platform to the same measures from the same samples assessed by clinical chemistry measurements ( Figure 2). Clinical chemistry measurements were completed at the BRI laboratory (fasting glucose) or Glasgow Royal Infirmary (lipids). Glucose was measured using a glucose oxidase method that does not cross-react with insulin. Total cholesterol, HDLc and triglycerides were measured following the standard Lipid Research Clinics Protocol using enzymatic reagents. LDLc was estimated from total cholesterol, HDLc and triglycerides (LDLc = [Total cholesterol in mmol/l] – [HDLc in mmol/l] – [Triglycerides in mmol/l ÷ 2.2]). The correlation between fasting glucose measured by clinical chemistry and by NMR was 0.73 and for all four lipids was between 0.85 and 0.93, with the intercepts of the regression line close to zero for HDLc, LDLc, and triglycerides, but higher for glucose (1.85) and total cholesterol (1.21). This suggests that the NMR platform systematically underestimates glucose and total cholesterol levels. However, the high levels of correlation, particularly for the lipid measures, is reassuring and suggests association analyses would have validity. To further test the validity of the NMR measures, we compared associations of maternal early pregnancy body mass index (BMI), treated as an exposure, with fasting glucose, and the four lipid measures from clinical chemistry and NMR as the outcome. We also compared associations between the five metabolic measures (from clinical chemistry and NMR) as exposures, with hypertensive disorder of pregnancy (HDP; either gestational hypertension or pre-eclampsia, defined on the basis of international criteria applied to all measures of blood pressure and proteinuria extracted from clinical records) ^12^ as the outcome. Associations of BMI with the five outcomes were directionally consistent between clinical chemistry and NMR measurements. However, the NMR associations were weaker (closer to the null) and there were clear differences in magnitudes of association between the two methods for the associations of BMI with glucose and HDLc ( Figure 3A). By contrast, results were concordant between the two methods for the associations of metabolites with odds ratios of HDP ( Figure 3B).

**Figure 4.**
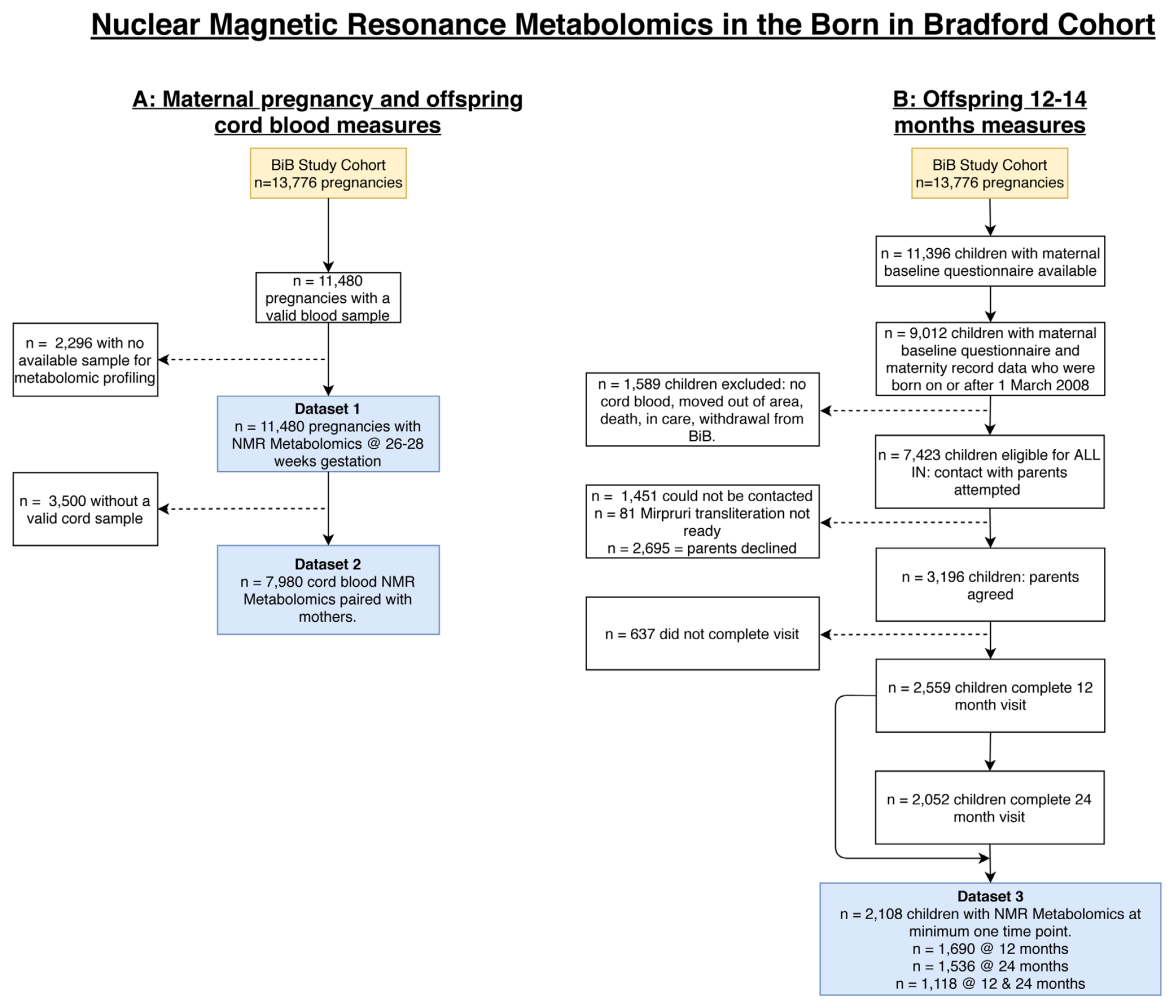
Illustrating the flow of participants into the NMR datasets in the Born in Bradford cohort. Figure **4A** shows the maternal pregnancy (Dataset 1: NMR metabolomics at 26–28 weeks’ gestation) and offspring cord blood samples (Dataset 2: NMR metabolomics taken from the umbilical vein shortly after delivery). Figure **4B** shows the offspring 12–24 months NMR metabolomic sample (Dataset 3). Abbreviations: NMR, nuclear magnetic resonance; BiB, Born in Bradford; ALL IN, Allergy and Infection study.

***Participant selection and characteristics of those with NMR data.*** In this subsection, we present flow charts to illustrate selection and inclusions into the NMR participant groups ( Figure 4) and describe participant characteristics for the BiB NMR datasets ( Table 2). All three of the samples of BiB participants with NMR data (maternal pregnancy N = 11,480, offspring cord blood N = 7,980, and offspring 12–24 months N = 2,108) had very similar distributions of maternal age, parity, early pregnancy BMI, residential area deprivation, offspring sex and birth weight to those seen in the whole cohort of 13,776 participants ( Table 2).

**Table 2.** Participant characteristics for NMR datasets in the BiB cohort.

		Maternal pregnancy NMR dataset (n=11,480)	Offspring cord blood NMR dataset (n=7,890)	Offspring 12- or 24- months NMR dataset (n = 2,108)	BiB cohort (n=13,776)
**Characteristics**	**Unit / Category**				
***Maternal Age***	Years *Missing*	27.3 (5.6) 410 (3.6)	27.5 (5.6) 627 (7.9)	28.3 (5.7) 60 (2.9)	27.3 (5.6) 1445 (10.5)
***Maternal Parity***	Nulliparous Multiparous *Missing*	4310 (37.5) 6428 (55.9) 742 (6.5)	2765 (36.6) 5125 (65.0) 344 (4.4)	819(39.9) 1,233(58.4) 56 (2.7)	5101 (37.0) 7773 (56.4) 902 (6.5)
***Maternal BMI***	kg/m ^2^ *Missing*	26.1 (5.7) 2160 (18.8)	26.2 (5.7) 1464 (18.5)	26.2(5.5) 106 (5.0)	26.0 (5.7) 3281 (23.8)
***Maternal ethnicity***	White British Pakistani Other *Missing*	4268 (37.2) 4995 (43.5) 1887(16.4) 330 (2.4)	2902 (37.7) 3596 (46.7) 1206 (15.7) 186 (2.4)	769 (49.7) 1048 (49.7) 291 (13.8) 0	5055 (37.8) 6088 (45.5) 2223 (16.6) 410 (3.0)
***Index of multiple*** ***deprivation***	Quintile 1 (most deprived) Quintile 2 Quintile 3 Quintile 4 Quintile 5 (least deprived) *Missing*	6646 (65.9) 1830 (18.2) 1124 (11.2) 306 (3.0) 173 (1.7) 1401 (12.2)	4439 (65.8) 1220 (18.1) 778 (11.5) 187 (2.8) 118 (1.8) 1148 (14.6)	1400(66.4) 355 (16.8) 248 (11.8) 70 (3.3) 34(1.6) 1 (0.0)	7566 (66.4) 2052 (18.0) 1250 (11.0) 334 (2.9) 190 (1.7) 2384 (17.3)
***Offspring sex***	Male Female *Missing*	5705 (49.7) 5420 (48.7) 355 (3.1)	4095 (51.9) 3795 (48.1) 3 (0.0)	1065(50.2) 1029(48.1) 14 (0.7)	6891 (50.0) 6470 (48.4) 415 (3.0)
***Birth weight***	Grams *Missing*	3226 (565) 356 (3.1)	3266 (522) 4 (0.1)	3224 (558) 14 (0.7)	3216 (565) 416 (3.0)

Data are means ± SD or n (%) unless stated. ^ gestational age in weeks presented for columns 1, 2 and 3. Offspring age in weeks presented for column 4. Abbreviations: NMR, nuclear magnetic resonance; BiB, Born in Bradford; BMI, body mass index; kg, kilogram; IMD, index of multiple deprivation (taken from 2010 national quintiles). There were 9 ethnic groups, of which White British and Pakistani were the main homogeneous groups. The 'Other' ethnicity category comprises: White Other, Mixed-White and Black, Mixed-White and South Asian, Black, Indian, Bangladeshi or Other ethnicity.

### Mass spectrometry metabolomics

***Mass spectrometry methods.*** The untargeted MS metabolomics analysis of over 1,000 metabolites was performed at Metabolon, Inc. (Durham, North Carolina, USA). Samples were sent to Metabolon in two separate batches. Dataset 1 was completed in December 2017 and consisted of 1,000 maternal pregnancy samples and 1,000 offspring paired cord blood samples. Dataset 2 was completed in December 2018 and consisted of 2,000 maternal pregnancy samples.

At Metabolon, samples were managed by a laboratory information management system and were kept at -80°C. Recovery standards were added to samples prior to monitor the extraction process. To remove proteins, dissociate small molecules bound to proteins, disassociate molecules trapped in the precipitated protein matrices, and to recover chemically diverse metabolites, proteins were precipitated with methanol under vigorous shaking for 2 min (Glen Mills GenoGrinder 2000) followed by centrifugation. The resulting extract was divided into five fractions: two for analysis by two separate reverse phase ultra-high-performance liquid chromatography-tandem mass spectrometry (UPLC-MS/MS) methods with positive ion mode electrospray ionization (ESI), one for analysis by reverse phase UPLC-MS/MS with negative ion mode ESI, one for analysis by hydrophilic interactive liquid chromatography (HILIC)/UPLC-MS/MS with negative ion mode ESI, and one sample was reserved for backup. Samples were placed on a TurboVap® (Zymark) to remove the organic solvent. The sample extracts were stored overnight under nitrogen before preparation for analysis. The four independent UPLC-MS/MS methods provide relative quantification of >1,000 metabolites in 10 key classes: amino acids, carbohydrates, lipids, nucleotides, microbiota metabolism, carbon metabolism, energy, cofactors & vitamins, xenobiotics, and unidentified metabolites. A list of metabolites defined in each of the datasets can be found in the *Extended data* ^11^. Metabolite concentrations were quantified using area-under-the-curve of primary MS ions and were expressed as the means of the medians (MoM) value for all batches processed on the given day.

***Validation of the MS platform.*** Details of the Metabolon QC procedures and data quality for the Metabolon BiB datasets are described in the reports found in the *Extended data* ^11^. In brief, procedures were conducted to: (i) assure that all aspects of the Metabolon process are operating within specifications, (ii) assess the effect of a non-plasma matrix on the Metabolon process and distinguish biological variability from process variability, (iii) assess the contribution to compound signals from the process (using Process Blank) and (iv) segregate contamination sources in the extraction (using Solvent Blank).

**Figure 5.**
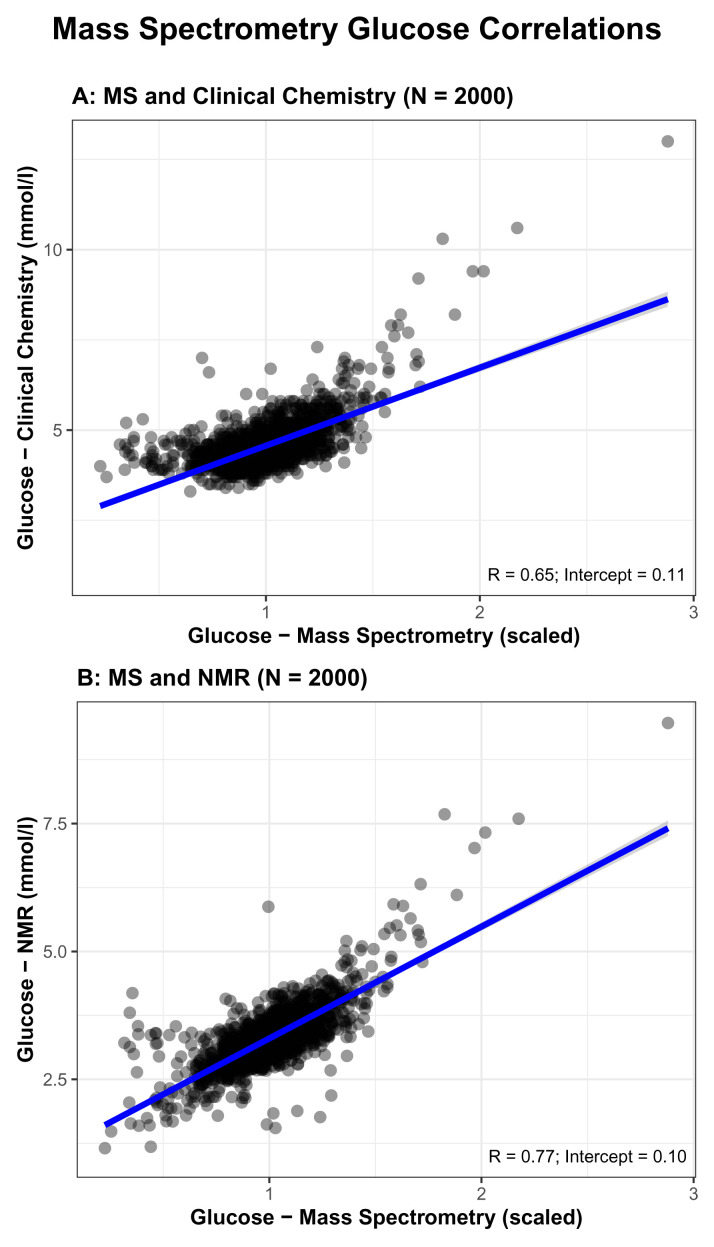
Comparisons of glucose concentrations for Metabolon mass spectrometry (MS) with routine glucose oxidase ( **5A**) and Nightingale Health© nuclear magnetic resonance (NMR) ( **5B**). R = Pearson correlation coefficient.

As an additional data QC, we explored correlations between MS and both NMR and clinical chemistry fasting glucose measures (glucose is the only common trait we have data on for MS, NMR, and clinical chemistry). Pearson’s correlation coefficient comparing MS to clinical chemistry (0.65) was modest and lower than that for NMR (0.73, see above and Figure 2) and the intercept was 0.11 ( Figure 5A). Correlation between Metabolon and NMR was higher (0.77) and the intercept was 0.10 ( Figure 5B).

**Figure 6.**
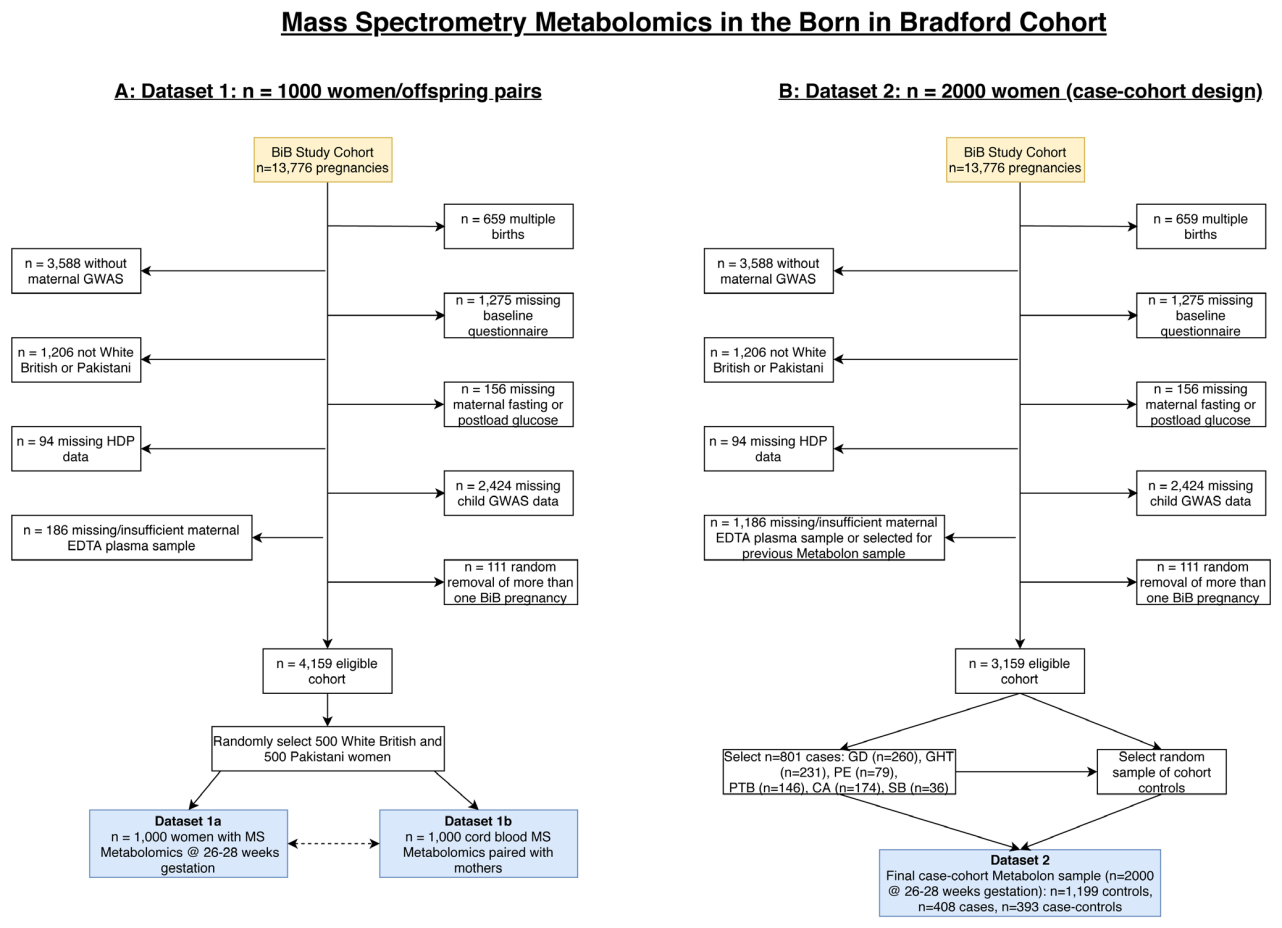
Illustrating the flow of participants into the Metabolon datasets in the Born in Bradford cohort. Figure **6A** shows dataset 1 which includes 1,000 pregnancies and infants with MS metabolomics during pregnancy (26–28 weeks’ gestation (dataset 1a)) and in cord blood (dataset 1b). Figure **6B** shows dataset 2 which includes 2,000 pregnancies (26–28 weeks’ gestation) with MS metabolomics within a case-cohort design. Abbreviations: MS, mass spectrometry; BiB, Born in Bradford; GWAS, genome wide association study; EDTA, ethylenediaminetetraacetic acid; HDP, hypertensive disorders of pregnancy; GD, gestational diabetes; GHT, gestational hypertension; PE, pre-eclampsia, PTB, preterm birth; CA, congenital anomaly; SB, still birth.

***Participant selection and characteristics of those with MS data.*** The flow of participants into the MS datasets are illustrated in Figure 6, and the characteristics of participants included in the two MS datasets provided in Table 3. Only women of either Pakistani or White British ethnic background were included in the MS datasets because, due to cost, we were only able to do this on a subset of the cohort. As these two groups represent ~85% of BiB it was felt the numbers for any other group would be too small for meaningful analyses. In Dataset 1, 1,000 women were selected on the basis that they had stored fasting plasma, a useable cord blood sample, genome wide data on both mother and offspring and were either of White British or Pakistani origin ( Figure 6A). Following these inclusions, 500 women were selected at random from each ethnic group (White British and Pakistani). In Dataset 2, a case-cohort design was used ^13, 14^. A case-cohort design consists of a cohort with an over-sampling of all cases. The BiB case-cohort consists of 2,000 women (only pregnancy samples were assayed in Dataset 2). As with Dataset 1, women were selected based on certain characteristics shown in Figure 6B, including that they had not already had Metabolon MS analyses. From those who fulfilled these pre-specified criteria, six groups of cases were selected: women with (a) gestational diabetes; (b) gestational hypertension; (c) pre-eclampsia; (d) preterm birth; (e) congenital anomaly; (f) stillbirth. In total, 801 women had experienced one or more of these conditions. Having selected all cases these were then replaced into the eligible cohort and a sub-cohort of 1,199 women were randomly selected from the eligible cohort. Thus, the comparison group in this case-cohort study is representative of the eligible cohort (i.e. the cohort comparison group includes some of the cases in proportions that would reflect the whole cohort). The final BiB case-cohort sample consists of three groups ( Figure 6B): 1) selected as comparison group (N = 1,199), 2) selected as cases only (N = 408), and 3) selected as a case and control (N = 393). The comparison group in any analyses will vary depending on the research question.

**Table 3.** Participant characteristics of the mass spectrometry datasets in the BiB cohort.

		Dataset 1 (N = 1,000 mother/child pairs)	Dataset 2 case-cohort ^a^ (N = 2,000)	Dataset 2 random cohort sample only ^b^ (N = 1,199)	BiB cohort (N = 13,776)
**Characteristics**	**Category**	-	-		-
***Maternal age***	Years	27.5 (5.7)	27.5 (5.7)	26.91 (5.5)	27.3 (5.6)
	*Missing*	0 (0)	0 (0)	0 (0.0)	1445 (10.5)
***Maternal parity***	Nulliparous Multiparous *Missing*	359 (37.0) 611 (61.1) 30 (3.0)	745 (37.3) 1213 (60.1) 42 (2.1)	433 (37.4) 725 (60.5) 41 (3.4)	5101 (37.0) 7773 (56.4) 902 (6.5)
***Maternal BMI***	(kg/m ^2^) *Missing*	26.7 (6.0) 36 (3.6)	26.8 (5.9) 97 (4.9)	25.9 (5.4) 60 (5.0)	26.0 (5.7) 3281 (23.8)
***Maternal ethnicity***	White British Pakistani Other *Missing*	500 (50.0) 500 (50.0) 0 0	933 (46.7) 1067 (53.4) 0 0	537 (44.8) 662 (55.2) 0 0	5055 (37.8) 6088 (45.5) 2223 (16.6) 410 (3.0)
***Index of multiple*** ***deprivation***	Quintile 1 (most deprived) Quintile 2 Quintile 3 Quintile 4 Quintile 5 (least deprived) *Missing*	656 (65.6) 175 (17.5) 112 (11.2) 38 (3.8) 19 (1.9) 0 (0)	1340 (67.0) 358 (17.9) 212 (10.6) 53 (2.6) 37 (1.8) 0 (0)	823 (68.6) 203 (16.9) 123 (10.3) 31 (2.6) 19 (1.6) 0 (0)	7566 (66.4) 2052 (18.0) 1250 (11.0) 334 (2.9) 190 (1.7) 2384 (17.3)
***Offspring sex***	Male Female *Missing*	512 (51.2) 488 (48.8) 0 (0)	1053 (52.7) 947 (47.3) 0 (0)	625 (52.1) 574 (47.9) 0 (0)	6891 (50.0) 6470 (48.4) 415 (3.0)
***Offspring*** ***birthweight***	Grams *Missing*	3304 (517) 0 (0)	3232 (574) 1 (0)	3318 (486) 0 (0)	3216 (565) 416 (3.0)

a This column comprises of the full case-cohort dataset of 2,000 pregnancies. This includes 801 selected cases and the 1,199 random cohort. b This column includes only the 1,199 random cohort to compare to the full case-cohort with the selected cases. Data are means ± SD or n (%) unless stated. Abbreviations: BiB, Born in Bradford; BMI, body mass index; kg, kilogram; IMD, index of multiple deprivation (taken from 2010 national quintiles). There were nine ethnic groups, of which White British and Pakistani were the main homogeneous groups. The 'Other' ethnicity category comprises: White Other, Mixed-White and Black, Mixed-White and South Asian, Black, Indian, Bangladeshi or Other ethnicity.

For the MS dataset, researchers are given the option of using the ‘raw’ data from Metabolon or a quantified (scaled) data set, in which missing data have been imputed and the multiple of median values transformed to standard deviation- (z-) scores (by subtracting the sample mean value for each metabolite from the participant value and then dividing by the sample standard deviation for that metabolite). This transformation helps overcome the problem of high missing data in metabolomics ^15^. This cohort for MS profiling were sampled on their ethnicity. It is almost 50% White British and Pakistani (there are slightly more Pakistani women in Dataset 2), as opposed to around 15% of the whole BiB cohort not belonging to either of these ethnic groups. However aside from this, the sample is representative of the whole cohort ( Table 3).

## Overlap between metabolomics datasets

**Figure 7.**
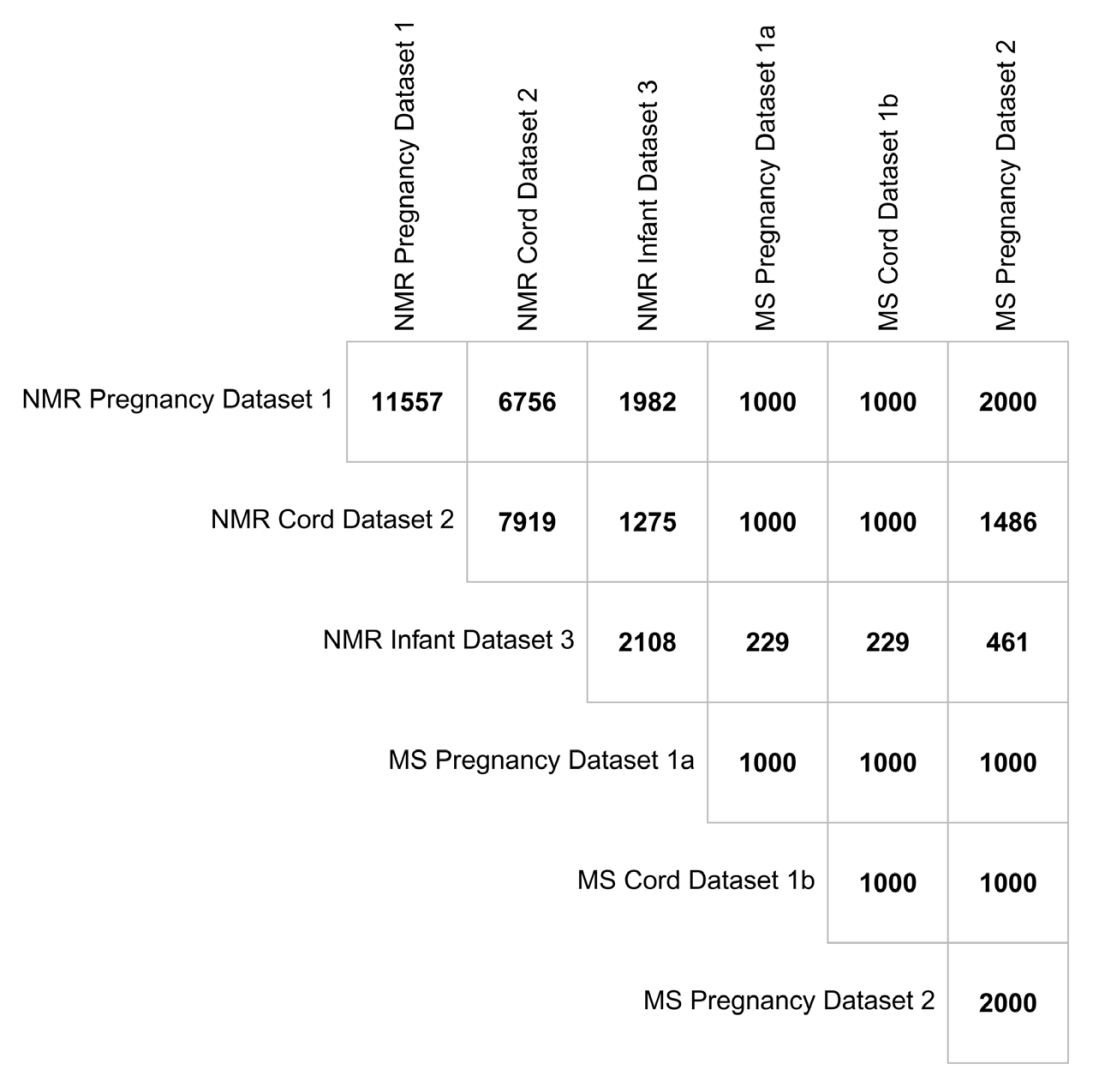
Showing the overlap between the metabolomic datasets in the Born in Bradford cohort presented at the offspring level. Abbreviations: NMR, Nuclear magnetic resonance; MS, mass spectrometry.

Having participants in multiple datasets (i.e. maternal pregnancy, offspring cord, offspring 12–24 months) and across the two metabolomic platforms provides scope for unique research opportunities. Figure 7 illustrates the overlap between BiB metabolomic datasets. The numbers are all based around the offspring, for example the number of maternal pregnancy metabolite data in any cell refer to the number of offspring who have a mother with those samples. There were 11,557 children from 11,480 pregnancies whose mothers had a pregnancy NMR sample. Of these, 6,756 children also had a cord blood sample and 1,981 had at least one measurement from either the 12- or 24-month ALL IN subsample. All the mothers with a pregnancy MS sample (from either the first or second dataset) also have an NMR sample. There were 7,919 children in total with an NMR sample in cord blood with 1,275 of these also having at least one measure from the 12- or 24-month subsample. Of those with NMR cord blood data, 2,486 had a mother with MS pregnancy data (from either the first or second dataset) and 1,000 have MS cord blood data. There were 2,108 children with at least one NMR measure at either the 12- or 24-months assessment and of these, 690 have a mother with MS metabolite measures in pregnancy data (from either dataset) and 229 have MS cord blood data. Although the exclusion criteria for MS dataset 2 was no prior MS metabolomics ( Figure 6), there was one mother with MS metabolomics in both datasets from different pregnancies.

## Using the BiB metabolomic data, including a summary of published, ongoing and future research using these data

The current BiB metabolite data have been quantified on blood samples collected during pregnancy, cord blood at birth and in the offspring at 12- and 24-months. These are critical time periods for life-course research and the combination of these data with large amounts of genomic, epigenomic, social and health data makes BiB a platform which provides scope for unique research opportunities.

### Issues for data users

*1. Batch effects*: The quantified NMR metabolites that have been measured in BiB are represented in clinically meaningful units, so can be compared to results from other studies. By contrast the Metabolon MS metabolites are quantified in relative abundance i.e. in relation to other quantified MS measurements that were run on the same day. The MS Dataset 1 and Dataset 2 were obtained ~2 years apart and have been normalized to different references, so are not directly comparable. For example, the value of a specific metabolite from a maternal pregnancy sample in Dataset 1 compared to the same metabolite in Dataset 2 may differ because they are from different batches. Because of the different selection process for the two datasets (Dataset 1 is paired pregnancy-offspring cord blood samples and Dataset 2 has a case-cohort sampling frame) it is not possible to normalize them to the same reference. We recommend running analyses separately in each of the two datasets and comparing results, then meta-analyse if appropriate. In principal components analyses, 37 of the 1,000 women with a pregnancy sample in Dataset 1 of MS data had notably different values to those in the remaining 963 women, which appeared to be a batch effect (see Figure in *Extended data* ^11^). This is a new finding and in previous analyses using these data we have not treated these 37 women differently. However, for future analyses we would recommend researchers consider running analyses with all women and in a sensitivity analysis with these 37 women removed.

*2. Comparisons with clinical chemistry measurements*: We have illustrated above strong correlations between glucose and lipids measured using clinical chemistry and the NMR platform. We found weaker (though directionally consistent) associations of BMI with these outcomes measured using NMR compared to those with clinical chemistry. In a second example, results were consistent between the two methods for the associations of pre-eclampsia with glucose and lipids. Researchers considering using these data might want to check for consistency with associations using the clinical chemistry measurement available in BiB. For the MS data we were only able to explore correlations with glucose and found this to be high between clinical chemistry and MS.

### Summary of published research using the BiB metabolomics data

We undertook a collaboration between BiB and the UK Pregnancies Better Eating and Activity Trial (UPBEAT), a randomised control trial of obese pregnant women (BMI ≥ 30kg/m ^2^) ^16^. We found evidence that maternal pregnancy NMR samples can improve prediction of pregnancy related disorders ^16^. The prediction models consisting of NMR-derived metabolomics and established risk factors (maternal age, smoking, BMI, ethnicity, and parity) performed better than established risk factors alone for gestational diabetes, hypertensive disorders of pregnancy, small/large for gestational age but not preterm birth in BiB. We found directionally consistent, but attenuated, results in UPBEAT. The attenuated results in that validation sample may reflect the differences between the studies, model overfitting in BiB or both.

In other work, we have also shown that that the distributions of most of the NMR metabolic measures differed by ethnicity ^17^. White European women had higher levels of most lipoprotein subclasses, cholesterol, glycerides and phospholipids, monosaturated fatty acids, and creatinine but lower levels of glucose, linoleic acid, omega-6 and polyunsaturated fatty acids, and most amino acids, compared with South Asian women. This suggests a more lipidomic response to pregnancy in White Europeans and a stronger glycaemic response in South Asian women. Higher BMI and having gestational diabetes were associated with higher levels of several lipoprotein subclasses, triglycerides, and other metabolites in both groups but with evidence of weaker magnitudes of association for most of these in the South Asian women.

In recent collaborations between the BiB cohort and the Pregnancy Outcome Prediction study (POPs) using Metabolon MS data, we have found evidence that 4’-hydroxyglutamate improves prediction of pre-eclampsia compared to clinical risk factors alone ^3^ and that a ratio of four metabolites (1-(1-enyl-stearoyl)-2-oleoyl-GPC, 1,5-anhydroglucitol,5α-androstan-3α,17α-diol disulfate and N1,N12-diacetylspermine) together with the sFlt-1:PIGF ratio is a better predictor of foetal growth restriction/small for gestational age than sFlt-1:PIGF alone ^4^. Initial associations in POPs, a nulliparous, largely White European, affluent cohort from the South East of England, were validated in BiB. As we have outlined, BiB is a cohort of mixed ethnic background, with high levels of deprivation and including both nulliparous and multiparous women. The consistency of associations between POPs and BiB suggests that the prediction accuracy may be widely generalisable and that the metabolites predicting these outcomes may be causally related to them.

Furthermore, combining the MS metabolomics with genomic sequence data has enabled the establishment of metabolomic consequences of loss of functional rare variants in autozygous individuals and the health effects of this loss of function ^18^. This has supported the development of the drug *lumasiran* for a rare kidney disease ^19^.

### Ongoing and future research

Ongoing work using both the NMR and MS metabolomics data will explore how the pregnancy metabolic environment relates to fetal growth (using repeat ultrasound scan measures and birth weight), preterm delivery, and congenital heart disease. Potential causal effects in these studies will be explored where possible by replication, the use of Mendelian randomization (MR) and triangulation with other types of data and study designs. In ongoing work, we are using data from both MS datasets to evaluate whether MS-derived metabolomics are better predictors of gestational diabetes, hypertensive disorders of pregnancy, small and large for gestational age and preterm birth, than risk factors alone (with external validation being undertaken in the POPs cohort). By combining both NMR and MS data, we are exploring the relationships between maternal pregnancy metabolites and their offspring cord blood metabolites.

BiB also contributes to metabolomic studies that are being undertaken by large collaborative efforts. This includes the European H2020 funded LifeCycle project ^20^, in which we are exploring exposure to maternal hypertensive disorder of pregnancy, gestational diabetes, small and large for gestational age and preterm delivery on offspring subsequent metabolic profile. In the Consortium of Metabolomics Studies (COMETS) ^2^ there are ongoing projects including trans-ethnic genome-wide association analyses (GWAS), and exploring effects of BMI, smoking, dietary patterns and hypertension on maternal metabolomic profiles.

## Discussion and future directions for metabolomic analyses in BiB

In this data note we have described multiple datasets with NMR and MS metabolomic measures in the BiB cohort. The wealth of metabolomics data available in BiB provides opportunities for addressing a range of research questions. In this section, we discuss the strengths and limitations of the data, together with some of our insights for using these data. We also provide information on plans for future measurements of metabolomics in BiB.

A key strength of these datasets is that they are based within a cohort that has very detailed information on 13,776 pregnancies. This includes detailed socioeconomic, education, cognitive, and mental and physical health data. We have OGTT results and fasting pregnancy blood samples on most (83%) of the mothers, genomics (genome wide and sequence) data and epigenomics data in maternal pregnancy and offspring cord blood. Few studies have pregnancy metabolomics data or OGTT data in numbers of this size. We can look at metabolomics and its role in prediction of adverse pregnancy/perinatal outcomes and health and development in children. BiB has large numbers of South Asian and White European families, residing in a city with high levels of socioeconomic deprivation. The ethnic diversity allows us to try and understand ethnic differences in the developmental origins of disease, for example, why South Asian populations have a higher risk of type two diabetes and coronary heart disease.

Having access to two metabolomics profiles (NMR and MS) is beneficial. The NMR platform mostly consists of lipids and lipoproteins, but also provides quantified fatty acids, amino acids, glycolysis metabolites, ketone bodies and glycoprotein acetyl (an inflammatory marker). It provides considerably more information than clinical chemistry measures that are conventionally measured in cohorts (e.g. glucose, total cholesterol, LDLc, HDLc and triglycerides) and at not much more cost (~£20 per sample). As a result, we have been able to obtain these data on large numbers of women in pregnancy, offspring cord blood and in samples taken in offspring at 12- and 24- months assessments. By contrast, the MS data covers more of the metabolome, including being able to assess energy metabolism (which might be important in pregnancy) and markers of medications such as paracetamol. However, it is more expensive (~£80-£200, depending on how many samples are assayed at a time). By having access to both datasets here, we can have broader coverage of the metabolome ^21^. There are potential uses for both platforms – ranging from disease prediction to causal analyses using methods such as MR ^22^. Both platforms have been used in previous GWAS of metabolites ^23^. As such, BiB could be used to explore whether genetic instruments from GWAS can be related to NMR or MS metabolites in pregnancy.

Access to this unique metabolomic data is a big advantage in BiB. However, we have been unable to validate findings in external cohorts. The work described above cannot be replicated because we cannot find other independent studies with relevant data ^17^. We hope that this data note will encourage other studies to collect similar data in pregnancy, offspring cord blood, and in mothers and offspring postnatally throughout their life-course.

There are some additional important limitations of the data to consider. The impact of these limitations will depend on the research question. All the metabolomics datasets were collected on subsamples ranging from 11,480 with maternal pregnancy NMR samples (83% of the eligible 13,776 participants) to 1,000 (7%) with MS cord blood samples. Smaller sample sizes may be statistically inefficient in some analyses and the selection processes ( Figure 4 and Figure 6) may result in selection bias in some analyses. It is notable and provides some reassurance that, even for the smaller samples, distributions of most characteristics are similar between participant groups with different types of metabolomics at different time points and the whole cohort ( Table 2 and Table 3). As Metabolon MS data have been collected only on White British and Pakistani women it cannot be assumed that analyses with these data would generalize to other ethnic groups. BiB cohort participants were largely recruited at the OGTT (with a small number recruited after that). This was opportunistic as we had no funding for initiating the cohort. After consultation with the community and health care providers, we established that this would be a suitable time to obtain consent, interview pregnant women and collect a fasting blood sample for research. However, it means that we are likely to have missed women who did not attend the OGTT and were not captured later in pregnancy or at delivery, and those who delivered pre-term before they attended their OGTT. We have previously compared BiB participants to non-BiB births occurring between 2007–2011 ^8^. Summary data from obstetric and delivery records were obtained for 11,761 non-BiB births, which would include some who moved to Bradford shortly before delivery (and would not have been eligible to recruit). The comparison showed a small number of differences. BiB participants were less likely to include younger mothers (age 20–24 years) and had a higher proportion of South Asian and nulliparous mothers. There were differences in gestational age and preterm delivery that reflected recruiting BiB participants relatively late in pregnancy ^8^. This selection on gestational age may introduce selection bias in some BiB analyses, including those using the metabolomics data described here.

A limitation is that BiB only has pregnancy metabolomics at a single time point and does not have pre-pregnancy measurements. Previous research suggests metabolites change upon becoming pregnant and then revert to pre-pregnancy levels ^5^ and that they change during pregnancy ^24^. Earlier measures would be valuable for prediction of future adverse outcomes to enable earlier antenatal monitoring and intervention.

This data note has focused on metabolomics data that have been quantified by high throughput commercial platforms (Nightingale Health© NMR and Metabolon MS). On a small subsample of BiB participants (N = 199) NMR urine and serum MS blood metabolites have been quantified at Imperial College, London, as part of the HELIX collaboration. HELIX aims to identify the human exposome in pregnancy and childhood. Metabolite measurements were undertaken alongside similar subsamples from five other cohorts (total N = 1,192). In all six cohorts, samples were from children aged between 6–11 years (BiB participants were mean age 6.6 years). 44 urine metabolites (24 semi-quantified) and 188 serum (56 fully quantified) metabolites were measured. We have not described these metabolomics datasets here as the assays are unique to a small subgroup of BiB participants and any research on these participants is best done together with the other HELIX cohort subgroups on whom the same metabolomic data obtained at the same time and using the same methods is available. Further information about the samples and methods used can be found elsewhere ^25^.

Up until March 2020, we were undertaking a follow-up of BiB parents and offspring, including collecting further blood samples with funding available to complete the NMR analyses on offspring and parent serum/plasma collected at this follow-up. However, that follow-up stopped on the 16 ^th^ March 2020 when restrictions on normal life due to the COVID-19 pandemic began in the UK. At the time of submitting this paper we do not know when face-to-face data collection will be possible to start again and what the best plans would be for further blood sample collection. At the relevant time we will discuss different potential scenarios for completing that planned follow-up with our scientific advisory groups. Whatever the decision, we should have some participants with serum/plasma NMR measures collected ~8–9 years after birth. We are also planning to measure metabolites on the available maternal pregnancy urine samples. Urine metabolites often provide a more accurate measure of dietary intake and medicine use than serum/plasma measures and would be a valuable addition to the existing datasets described here. Any new data will be made available to the wider research community.

## Data availability

### Underlying data

Scientists are encouraged to make use of the BiB data, which are available through a system of managed open access.

- Before you contact BiB, please make sure you have read our Guidance for Collaborators. Our BiB executive review proposals on a monthly basis and we will endeavour to respond to your request as soon as possible. You can find out about all of the different datasets which are available here. If you are unsure if we have the data that you need please contact a member of the BiB team ( borninbradford@bthft.nhs.uk).
- Once you have formulated your request please complete the ‘Expression of Interest’ form available here and email the BiB research team ( borninbradford@bthft.nhs.uk).
- If your request is approved, we will ask you to sign a collaboration agreement; if your request involves biological samples, we will ask you to complete a material transfer agreement.

### Extended data

Open Science Framework: Metabolomics data in the Born in Bradford cohort. https://doi.org/10.17605/OSF.IO/YST7N ^11^.

This project contains the following extended data:

- BiB_MS_Dataset1_PCA_Plot.png (Figure showing principal component analysis of dataset 1)
- MS_Metabolite_Details.xlsx (Lists the names and details of all metabolites assessed by the Metabolon platform in the BiB MS dataset 1a (sheet 1) and BiB MS dataset 2 (sheet 2).
- MS_Quality_Report_Dataset1.pdf (QC report for MS dataset 1 from Metabolon)
- MS_Quality_Report_Dataset2.pdf (QC report for MS dataset 2 from Metabolon)
- NMR_Metabolite_Details.xlsx (Lists the names and units of all metabolic traits assessed by the NMR platform)
- NMR_Quality_Report_Cord.pdf (Summarizing quality observations in the NMR cord blood dataset)
- NMR_Quality_Report_Infant.pdf (Summarizing quality observations in the NMR infant dataset)
- NMR_Quality_Report_Pregnancy.pdf (Summarizing quality observations in the NMR pregnancy dataset)

Data are available under the terms of the Creative Commons Attribution 4.0 International license (CC-BY 4.0).
