# Supplementary material for: Metabolomics datasets in the Born in Bradford cohort
Source: Wellcome Open Res. 2021 Sep 10;5:264. Originally published 2020 Nov 5. [Version 2] doi: 10.12688/wellcomeopenres.16341.2 (PMC11109709; doi:10.12688/wellcomeopenres.16341.2)
Supplement: Peer review history

# Reviewer report: Reviewer response for version 2

**DOI:** 10.21956/wellcomeopenres.18489.r107903

**Published:** 2024-10-28

**Authors:** Simone Zuffa

**Affiliations:** 1 University of California San Diego, San Diego, California, USA

Taylor K., McBride M., et al. describe the collection rationale and methods for metabolomics data in the Born in Bradford (BiB) cohort. Blood samples were obtained from pregnant women, umbilical cords, and infants, and analyzed using NMR and MS. The authors highlight that the metabolomics data generated from this cohort can be analyzed alongside the extensive metadata and other omics data to address significant questions related to pregnancy and health development in both mothers and offspring.

 The manuscript is well-structured and provides a clear overview of the collection practices. However, a major limitation of the presented data is that it was collected by commercial companies, and the generated raw data is not available to the research community. The field of computational metabolomics has advanced significantly in the past decade, and many tools (e.g., GNPS ^1^, SIRIUS ^2^, MSNovelist ^3^, MassQL ^4^) are now available for the reanalysis of metabolomics data, particularly in the realm of untargeted mass spectrometry. Access to the raw mass spectrometry data in open-source formats, rather than just tabular data of extracted and annotated peak areas, could unlock the full potential of this cohort. This would enable investigations into novel classes of molecules, as well as drugs or environmental pollutants, which could be associated with the phenotypic information collected by the authors. I recommend that the authors engage with the companies to obtain the raw data and make it publicly available to researchers.

 Regarding other paired omics data available for the samples, it is unclear how many samples (from mothers and infants) have corresponding genomic data. From the Data Dictionary ( https://borninbradford.github.io/datadict/index.html), it also appears that proteomics and glycomics data are available. It would be helpful to include this information in the manuscript so that researchers have a clear overview of all the matching omics data available within the cohort.

 Other minor comments

1. The study website in page 3 link is broken.
2. Add a bit more information of how metabolomics can be targeted or untargeted.
3. The link “A list of metabolites defined in each of the datasets can be found  in the Extended data” at page 9 does not actually provide a list of the metabolites obtained via MS

Are sufficient details of methods and materials provided to allow replication by others?

Yes

Is the rationale for creating the dataset(s) clearly described?

Yes

Are the datasets clearly presented in a useable and accessible format?

Partly

Are the protocols appropriate and is the work technically sound?

Yes

Reviewer Expertise:

untargeted metabolomics, mass spectrometry, data science

I confirm that I have read this submission and believe that I have an appropriate level of expertise to confirm that it is of an acceptable scientific standard, however I have significant reservations, as outlined above.

## References

[1] : Sharing and community curation of mass spectrometry data with Global Natural Products Social Molecular Networking. *Nat Biotechnol* .2016;34(8) : 10.1038/nbt.3597 828-837 10.1038/nbt.3597 27504778 PMC5321674

[2] : SIRIUS 4: a rapid tool for turning tandem mass spectra into metabolite structure information. *Nat Methods* .2019;16(4) : 10.1038/s41592-019-0344-8 299-302 10.1038/s41592-019-0344-8 30886413

[3] : MSNovelist: de novo structure generation from mass spectra. *Nat Methods* .2022;19(7) : 10.1038/s41592-022-01486-3 865-870 10.1038/s41592-022-01486-3 35637304 PMC9262714

[4] : A Universal Language for Finding Mass Spectrometry Data Patterns. *bioRxiv* .2022; 10.1101/2022.08.06.503000 10.1101/2022.08.06.503000

# Reviewer report: Reviewer response for version 2

**DOI:** 10.21956/wellcomeopenres.18489.r79682

**Published:** 2024-05-21

**Authors:** Sandi Azab

**Affiliations:** 1 McMaster University, Hamilton, Ontario, Canada

Paper Review: Metabolomics Datasets in the Born in Bradford Cohort

 Thank you for the opportunity to review this manuscript submission. In this article, the authors describe the metabolomic data available in Born in Bradford (BiB), profiled during pregnancy in mothers, in cord blood and during early life in the offspring. The authors provide an overview of NMR and MS technologies used and the QC measures taken as well as the BiB cohort itself with some note on current and future metabolomics studies in bib.

 This manuscript is very well written and clearly explains the different metabolomics datasets in BiB. Thus, a definite strength of this study is its clarity and its overall overview and structure.

 However, the following remarks are to be considered:

Major comments:

- Following, although the authors mention Comments in the introduction, there is a lack of discussing the term “Metabolomic epidemiology” that was introduced by the Metabolomics Society Epidemiology Task Group (overlap with researchers behind comets). As the authors discuss outcomes, study design, and other epidemiologic concepts, it is important to refer to this paper for the opportunities and challenges that might arise when “marrying” metabolomics and epidemiology. [Metabolomics (2021) 17:45 https://doi.org/10.1007/s11306-021-01789-0] [Ref 1]

 

- The ethnic diversity in BiB is a point of strength. It will be valuable to note that in the abstract as well emphasize in the paper the scarcity of omics data in non-white ethnic populations and the need for ethnic expansion in the era of precision medicine. You can refer to this landmark Lancet series on precision medicine [Lancet Diabetes Endocrinol 2023; 11: 822–35] [Ref 2]

 

- I realize that the paper has been submitted in 2020 so I understand that it might not be feasible for the authors to update their literature review. However, I encourage to revise the following sentence based on existing birth cohorts with metabolomics data e.g., Canadian “NutriGen” birth cohort consortium and Australian “checkpoint”. *“However, we have been unable to validate findings* *in external cohorts. The work described above cannot be* *replicated because we cannot find other independent studies* *with relevant data* *19* *. We hope that this data note will encourage* *other studies to collect similar data in pregnancy, offspring* *cord blood, and in mothers and offspring postnatally* *throughout their life-course.” *

J Am Heart Assoc. 2019; [Ref 3];

 Sikorski C *et al 2022*  [Ref 4];

 Azab, S.(2023)[Ref 5] 

  

- Please add 1 sentence on the advantage of the case-cohort design; Why was it selected?

Minor comments:

- When referring to GDM, HDP etc., authors are advised to use the term “APOs” (Adverse Pregnancy outcomes) used in the 2024 American Heart Association Scientific Statement, *[Ref 6]*
- *“Previous work has shown that reproducibility in both serum and plasma is good.”* Ref needed.
- First paragraph in Intro needs several references.
- *“MS offers a truly untargeted approach.” *Please rephrase as MS can be used for either targeted or untargeted metabolomics depending on the method.
- *“However, MS only provides relative quantification based on peak area in these approaches without comparison to a metabolite reference standard.”* Please rephrase: While most high-throughput untargeted MS platforms provide relative peak areas, with available standards known metabolites can be quantified in absolute concentrations (mmol/l) using MS platform. See: “Shanmuganathan, M., Kroezen, Z., Gill, B. *et al.* The maternal serum metabolome by multisegment injection-capillary electrophoresis-mass spectrometry: a high-throughput platform and standardized data workflow for large-scale epidemiological studies. Shanmuganathan M et al, (2021). [Ref 7]” this reference also gives reference values in pregnancy for 50 metabolites or so using CE-MS.

 

- *“A list of metabolites defined in each of the datasets can be found in the* *Extended data11* *.” *As far as my skills allowed me, this links to a list of NMR data only and not MS variables. The reader cannot access a list of the 1000 molecules identified in the MS datasets *.*

 

- Second version of the Metabolon MS data: not clear what changed? can you clarify

 

- *“This suggests a more lipidomic pregnancy metabolic profile in White Europeans and a stronger glycemic metabolic profile in South Asian women.” *I would rephrase the “more lipidomic profile”! do you mean more perturbations in the lipidome/lipids in White Europeans?

 

- *“NMR urine and serum MS blood metabolites have been quantified at Imperial College, London, as part of the HELIX collaboration.” *Please change to NMR-quantified or identified metabolites; there are no NMR or MS metabolites per se.

 

- *However, that follow-up stopped on the 16 ^th^ March 2020 when restrictions on normal life due to the COVID-19 pandemic began in the UK. At the time of submitting this paper we do not know when face-to-face data collection will be possible to start again and what the best plans would be for further blood sample collection. At the relevant time we will discuss different potential scenarios for completing that planned follow-up with our scientific advisory groups. *It seems this sentence needs updating; Is there a plan forward? Or delete this sentence.

Thank you,

**Sandi Azab, MSc, RPh, PhD**

Are sufficient details of methods and materials provided to allow replication by others?

Yes

Is the rationale for creating the dataset(s) clearly described?

Yes

Are the datasets clearly presented in a useable and accessible format?

Partly

Are the protocols appropriate and is the work technically sound?

Yes

Reviewer Expertise:

Metabolomics; Bioanalytical chemistry; Mass spectrometry; Metabolomic epidemiology; DOhaD; birth cohorts; Cardiovascular disease

I confirm that I have read this submission and believe that I have an appropriate level of expertise to confirm that it is of an acceptable scientific standard, however I have significant reservations, as outlined above.

## References

[1] : A strategy for advancing for population-based scientific discovery using the metabolome: the establishment of the Metabolomics Society Metabolomic Epidemiology Task Group. *Metabolomics* .2021;17(5) : 10.1007/s11306-021-01789-0 45 10.1007/s11306-021-01789-0 33937923 PMC8605901

[2] : Lancet Diabetes & Endocrinology Commission on the Definition and Diagnosis of Clinical Obesity. *Lancet Diabetes Endocrinol* .2023;11(4) : 10.1016/S2213-8587(23)00058-X 226-228 10.1016/S2213-8587(23)00058-X 36878238

[3] : A Cross-Cohort Study Examining the Associations of Metabolomic Profile and Subclinical Atherosclerosis in Children and Their Parents: The Child Health CheckPoint Study and Avon Longitudinal Study of Parents and Children. *J Am Heart Assoc* .2019;8(14) : 10.1161/JAHA.118.011852 e011852 10.1161/JAHA.118.011852 31286813 PMC6662147

[4] : Serum metabolomic signatures of gestational diabetes in South Asian and white European women. *BMJ Open Diabetes Res Care* .2022;10(2) : 10.1136/bmjdrc-2021-002733 10.1136/bmjdrc-2021-002733 35450870 PMC9024260

[5] : Early sex-dependent differences in metabolic profiles of overweight and adiposity in young children: a cross-sectional analysis. *BMC Med* .2023;21(1) : 10.1186/s12916-023-02886-8 176 10.1186/s12916-023-02886-8 37158942 PMC10166631

[6] : Opportunities in the Postpartum Period to Reduce Cardiovascular Disease Risk After Adverse Pregnancy Outcomes: A Scientific Statement From the American Heart Association. *Circulation* .2024;149(7) : 10.1161/CIR.0000000000001212 e330-e346 10.1161/CIR.0000000000001212 38346104 PMC11185178

[7] : The maternal serum metabolome by multisegment injection-capillary electrophoresis-mass spectrometry: a high-throughput platform and standardized data workflow for large-scale epidemiological studies. *Nat Protoc* .2021;16(4) : 10.1038/s41596-020-00475-0 1966-1994 10.1038/s41596-020-00475-0 33674789

# Reviewer report: Reviewer response for version 2

**DOI:** 10.21956/wellcomeopenres.18489.r45817

**Published:** 2021-10-04

**Authors:** Ruifang Li-Gao

**Affiliations:** 1 Leiden University Medical Center, Leiden, The Netherlands

Thanks to the authors for the revisions and great work. I have no further comments to make.

Are sufficient details of methods and materials provided to allow replication by others?

Yes

Is the rationale for creating the dataset(s) clearly described?

Yes

Are the datasets clearly presented in a useable and accessible format?

Yes

Are the protocols appropriate and is the work technically sound?

Yes

Reviewer Expertise:

Metabolomics, genetics, twin/family study

I confirm that I have read this submission and believe that I have an appropriate level of expertise to confirm that it is of an acceptable scientific standard.

# Reviewer report: Reviewer response for version 1

**DOI:** 10.21956/wellcomeopenres.17967.r43230

**Published:** 2021-04-20

**Authors:** Mengna Huang

**Affiliations:** 1 Channing Division of Network Medicine, Department of Medicine, Brigham and Women’s Hospital and Harvard Medical School, Boston, MA, USA

Taylor *et al. *provided a comprehensive review of the metabolomic data currently available in the Born in Bradford (BiB) birth cohort, including those generated from NMR and MS platforms at different time points from 26–28 weeks gestation to ~24 months of the children. Details of the sample collection procedures and metabolomic profiling were described, including validation of some of the data using other assays. Overall all relevant information was very clearly presented in this data note.

 Below are some comments that may help the authors improve the manuscript:

1. Page 4 – the total number of pregnancies in BiB was greater than the number of women enrolled in the study, and the recruitment period spanned over four years. In Figures 4A and 6, mostly it was the number of pregnancies that were presented – were these singleton birth? Were there women who contributed samples from more than one pregnancy? Same question for cord blood. In figure 4B, were children with NMR metabolomics all from independent families? Such information will have implications in statistical analysis and should be clarified.
2. Page 4 – under “Pregnancy blood samples”, step 4 indicated that 2 vials were used, while step 6 says 3 aliquots. This seems inconsistent.
3. Page 7 – for validation of the MS platform, did the author conduct regression analysis with the MS glucose measure in association with BMI and HDP? This would of course be in a smaller sample size, but it would be interesting to see the results.
4. Page 10 – for the MS dataset, apart from the raw and scaled data sets from Metabolon, did the authors perform additional data processing (e.g. different imputation approach or log transformation)? There would usually be some metabolites with high missing percentages where imputing with the minimum (which I believe is what Metabolon usually does for imputation) would not be entirely appropriate for subsequent analysis. Is there any recommendation in terms of excluding them from subsequent analysis or applying a different analysis method?
5. Figure 7 – there should not be overlap between MS pregnancy dataset 1a and MS pregnancy dataset 2 according to descriptions on the bottom of Page 9?
6. Page 14 – the authors mentioned that differences by ethnicity in distributions of NMR data were observed, I wonder if differences in diet plays a role here. It’s not quite clear whether BiB collected dietary data from the main text?
7. The article provided a good overview of the data available in pregnancy other than the metabolomics data. How much and what kind of data would be available during early life for the children? From the description of HELIX on Page 16, it seemed like BiB children were followed up to a median age of 6.6 years, were all children followed-up? Or just those in the ALL IN sub-study?
8. Is there a plan to generate MS metabolomic data in the 12- and 24- months infant samples? Such data may complement the existing NMR data, and be important for studies like ALL IN.

Are sufficient details of methods and materials provided to allow replication by others?

Yes

Is the rationale for creating the dataset(s) clearly described?

Yes

Are the datasets clearly presented in a useable and accessible format?

Yes

Are the protocols appropriate and is the work technically sound?

Yes

Reviewer Expertise:

epidemiology, metabolomics, integrative omics, asthma

I confirm that I have read this submission and believe that I have an appropriate level of expertise to confirm that it is of an acceptable scientific standard, however I have significant reservations, as outlined above.

# Response

**Published:** 2021-09-06

**Authors:** Kurt Taylor

**Affiliations:** University of Bristol, UK

1. Page 4 – the total number of pregnancies in BiB was greater than the number of women enrolled in the study, and the recruitment period spanned over four years. In Figures 4A and 6, mostly it was the number of pregnancies that were presented – were these singleton birth? Were there women who contributed samples from more than one pregnancy? Same question for cord blood. In figure 4B, were children with NMR metabolomics all from independent families? Such information will have implications in statistical analysis and should be clarified.

**The reviewer makes a useful point, and we agree that having this information in the manuscript will be useful for readers and prospective users of the data. We have now added this information to Figures 4 and 6, which show that women with more than one pregnancy had one removed at random and those with multiple pregnancies were excluded.**

 2. Page 4 – under “Pregnancy blood samples”, step 4 indicated that 2 vials were used, while step 6 says 3 aliquots. This seems inconsistent.

**We thank the reviewer for pointing out this inconsistency, which we have now corrected by using only the term aliquot and clarifying the numbers:**

***“1) Storage racks were prepared.***

***2) Participant details were checked, making sure that both the BiB study ID and hospital number on the specimen bottles matched those on the participant tracking forms.***

***3) Tubes were centrifuged at 3500 rpm for 10 minutes at room temperature.***

***4) A 1 ml automatic pipette was used to aliquot samples into 1.5 ml aliquots (* 1-4 aliquots *dependent on sample volume).***

***5) Vials were labelled with appropriate BiB study labels and the duplicate barcode label was placed in the corresponding space marked on BiB tracking form.***

***6) Aliquots were then placed in racks in a -80°C freezer.”***

 3. Page 7 – for validation of the MS platform, did the author conduct regression analysis with the MS glucose measure in association with BMI and HDP? This would of course be in a smaller sample size, but it would be interesting to see the results.

**We thank the reviewer for pointing this out. We agree that it would be useful to include this. Please see response 2, to reviewer 1 above.**

 4. Page 10 – for the MS dataset, apart from the raw and scaled data sets from Metabolon, did the authors perform additional data processing (e.g. different imputation approach or log transformation)? There would usually be some metabolites with high missing percentages where imputing with the minimum (which I believe is what Metabolon usually does for imputation) would not be entirely appropriate for subsequent analysis. Is there any recommendation in terms of excluding them from subsequent analysis or applying a different analysis method?

**We agree with the reviewer. However, the data are provided by Metabolon following extensive quality control. We cannot give recommendations for analyses because it depends on the research question of the group using the data. For example, in some of our analyses we remove participants where there is little between person variation in a particular metabolite. We also convert xenobiotics where there is substantial missing data to binary variables (indicating exposure to e.g. a particular medication).**

 5. Figure 7 – there should not be overlap between MS pregnancy dataset 1a and MS pregnancy dataset 2 according to descriptions on the bottom of Page 9?

**We thank the reviewer for identifying this mistake. We have amended the figure to show the overlap of ‘0’.**

 6. Page 14 – the authors mentioned that differences by ethnicity in distributions of NMR data were observed, I wonder if differences in diet plays a role here. It’s not quite clear whether BiB collected dietary data from the main text?

**For clarity, we have added the following to the discussion section:**

***There is also scope to explore how diet could relate to the range of metabolomic measurements that BiB possesses. Further information on dietary variables can be found online in the BiB data dictionary https://borninbradford.github.io/datadict/.***

 7. The article provided a good overview of the data available in pregnancy other than the metabolomics data. How much and what kind of data would be available during early life for the children? From the description of HELIX on Page 16, it seemed like BiB children were followed up to a median age of 6.6 years, were all children followed-up? Or just those in the ALL IN sub-study?

**For clarity, we have added a sentence in the “ongoing and future research” section:**

***To date, there is no published work using the offspring metabolomics data. Researchers can find information on planned follow up data elsewhere, to understand whether these data could be useful to their ongoing or future research (* *https://doi.org/10.1186/s12889-019-7222-2* *).***

 8. Is there a plan to generate MS metabolomic data in the 12- and 24- months infant samples? Such data may complement the existing NMR data, and be important for studies like ALL IN.

**Currently there are no plans or funds to do this. Researchers who are interested in funding analyses on any available biosamples in BiB can submit an expression of interest to do so ( https://borninbradford.nhs.uk/research/how-to-access-data/). Because biosamples are finite these requests are assessed on several criteria, including the importance of the science, whether any existing assay results (including the metabolomic data described here) provide relevant data, whether other collaborators are already running similar assays and the volume required compared to current available sample volume.**

 -----------------------------------

**ADDITIONAL SECTION TO THE REVISED DATANOTE**

**Since submitting the data not new versions of the two Metabolon MS data have been derived. We have added a new section called “second version of the Metabolon MS data” in the “issues for data users” section. We describe why we obtained these new versions and some analyses of how they relate to the original versions.**

# Reviewer report: Reviewer response for version 1

**DOI:** 10.21956/wellcomeopenres.17967.r42218

**Published:** 2021-02-08

**Authors:** Ruifang Li-Gao

**Affiliations:** 1 Leiden University Medical Center, Leiden, The Netherlands

Taylor and colleagues described the metabolomics data profile in the Born in Bradford (BiB) cohort. Metabolomics data were generated from two different approaches, i.e. NMR-based target metabolomics by Nightingale platform and MS-based untargeted metabolomics by Metabolon platform. To address varied research questions, there are six metabolomics datasets including different sub-populations and time points of BiB cohort. Overall, the datasets clearly presented in a useable and accessible format. The amount of details of data generation and study protocol is sufficient. I have several minor questions for the authors:

1. Figure 2 shows the comparisons of measurements from NMR-based platform and clinical chemistry. In general, the correlations are high, between 0.73 and 0.93. However, in each measurement, there are “outliers” deviated from the diagonal. Are those measurements due to technical issues or participants’ biological characteristics? Do the authors recommend users of the datasets remove those outliers?
2. Figure 5 shows the comparisons of fasting glucose measures between MS-based platform and clinical chemistry. The correlation is modest (0.65). Is it possible to verify the direction and magnitude of associations by metabolon measurements, as the authors did for NMR-based measurements and were shown in Figure 3?      
3. In Figure 6B bottom part, “n=1,199 controls, n=408 cases, n=393 case-controls”. It is not very obvious where the numbers 408 and 393 coming from. Is it possible to explain this?
4. In Figure 7, it showed that the overlap between MS Pregnancy Dataset 1a and MS Pregnancy Dataset 2 is 1000. However, in Figure 6B, the authors mentioned that 1,186 removed due to missing or selected for previous metabolon sample. If the previous 1000 already removed, how could be the overlap is 1000?
5. On Page 13, the authors mentioned that 37 out of 1000 women in MS Pregnancy Dataset 1 had different values than the other 963 women. Could be the difference due to certain disease status?
6. On Page 14, the authors suggested that “a more lipidomic response to pregnancy in White Europeans and a stronger glycaemic response in South Asian women.” Since the cohort did not have measurements before pregnancy, it is not exact to use “response” here.
7. On Page 16, the authors mentioned that they did not have chance to collect blood samples before pregnancy, but previous research showed that “metabolites change upon becoming pregnant and then revert to pre-pregnancy levels [5] and that they change during pregnancy [24].”  Do the study consider to collect blood samples after pregnancy, as the proxy for before-pregnancy?

Are sufficient details of methods and materials provided to allow replication by others?

Yes

Is the rationale for creating the dataset(s) clearly described?

Yes

Are the datasets clearly presented in a useable and accessible format?

Yes

Are the protocols appropriate and is the work technically sound?

Yes

Reviewer Expertise:

metabolomics, genetics, twin/family study

I confirm that I have read this submission and believe that I have an appropriate level of expertise to confirm that it is of an acceptable scientific standard, however I have significant reservations, as outlined above.

# Response

**Published:** 2021-09-06

**Authors:** Kurt Taylor

**Affiliations:** University of Bristol, UK

2. Figure 2 shows the comparisons of measurements from NMR-based platform and clinical chemistry. In general, the correlations are high, between 0.73 and 0.93. However, in each measurement, there are “outliers” deviated from the diagonal. Are those measurements due to technical issues or participants’ biological characteristics? Do the authors recommend users of the datasets remove those outliers?

**This is a useful question and one that we cannot answer for certain. We have acknowledged the potential outliers within the revised manuscript and recommend that researchers carry out their own investigations into these, which will depend on their research question:**

***“It is evident from Figure 2 that there are outliers for some of the measures, notably for glucose, total cholesterol and triglycerides (Figures 2A, 2B, 2E, respectively). We would recommend for researchers using the data to consider these potential outliers before commencing analyses. Determining how to deal with outliers will depend on the research question and the personal preference of the research group undertaking analyses.”***

 2. Figure 5 shows the comparisons of fasting glucose measures between MS-based platform and clinical chemistry. The correlation is modest (0.65). Is it possible to verify the direction and magnitude of associations by metabolon measurements, as the authors did for NMR-based measurements and were shown in Figure 3?

**We thank the reviewer for pointing this out. We agree that it would be useful to include this. We have included these associations in Figure 3 to avoid having too many figures. We have also commented on the associations in text:**

***“Given the relatively modest correlation of glucose from the Metabolon mass spec analyses with the clinical chemistry levels on the same samples, we explored this further comparing results from two regression analyses – one of the differences in mean glucose per 1SD higher BMI (glucose as outcome) and one of the odds ratio for HDP per 1SD higher glucose (glucose as exposure).”***

 In Figure 6B bottom part, “n=1,199 controls, n=408 cases, n=393 case-controls”. It is not very obvious where the numbers 408 and 393 coming from. Is it possible to explain this?

**We have added the following to the Figure 6B legend:**

***“The 801 cases are split into “cases” and “case-controls” based on how many cases we would expect in a representative cohort (i.e. the case-controls). “Cases” should not be included in a comparator group for any analyses as we want the comparison group to be representative.”***

 3. In Figure 7, it showed that the overlap between MS Pregnancy Dataset 1a and MS Pregnancy Dataset 2 is 1000. However, in Figure 6B, the authors mentioned that 1,186 removed due to missing or selected for previous metabolon sample. If the previous 1000 already removed, how could be the overlap is 1000?

**We thank the reviewer for identifying this mistake. We have amended the figure to show the overlap of ‘0’.**

 4. On Page 13, the authors mentioned that 37 out of 1000 women in MS Pregnancy Dataset 1 had different values than the other 963 women. Could be the difference due to certain disease status?

**The reviewer makes a useful point. This was our initial thought when we first discovered the unusual values. We checked the samples against all the phenotype data and did not find anything to suggest that the differences were being caused by disease status, or any exposure such as smoking or BMI. We think that it is a batch effect, as mentioned within the manuscript:**

***“In principal components analyses, 37 of the 1,000 women with a pregnancy sample in Dataset 1 of MS data had notably different values to those in the remaining 963 women, which may be a batch effect (see Figure in* *Extended data* *^11^* *). This is a new finding and in previous analyses using these data we have not treated these 37 women differently. However, for future analyses we would recommend researchers consider running analyses with all women and in a sensitivity analysis with these 37 women removed.”***

 5. On Page 14, the authors suggested that “a more lipidomic response to pregnancy in White Europeans and a stronger glycaemic response in South Asian women.” Since the cohort did not have measurements before pregnancy, it is not exact to use “response” here.

**We agree with the reviewer. We have reworded this sentence:**

***This suggests a more lipidomic pregnancy metabolic profile in White Europeans and a stronger glycemic metabolic profile in South Asian women.***

 6. On Page 16, the authors mentioned that they did not have chance to collect blood samples before pregnancy, but previous research showed that “metabolites change upon becoming pregnant and then revert to pre-pregnancy levels [5] and that they change during pregnancy [24].”  Do the study consider to collect blood samples after pregnancy, as the proxy for before-pregnancy?

**The reviewer makes an interesting point. At the time that the COVID pandemic began, a follow-up of BiB participants was underway. The main focus was on the index children, with data largely collected in primary schools (including blood samples). However, some parents were recruited, and it is intended to run the NMR metabolites on those. The sample size will likely be smaller than anticipated.  We have noted this in the ‘Discussion and future directions for metabolomic analyses in BiB’ section and note there that we will update this data note regularly with any new metabolomics analyses.**

**----------------------------------**

**ADDITIONAL SECTION TO THE REVISED DATANOTE**

**Since submitting the data not new versions of the two Metabolon MS data have been derived. We have added a new section called “second version of the Metabolon MS data” in the “issues for data users” section. We describe why we obtained these new versions and some analyses of how they relate to the original versions.**
